# Characteristics and Management of Spinal Tuberculosis in Tuberculosis Endemic Area of Guizhou Province: A Retrospective Study of 597 Patients in a Teaching Hospital

**DOI:** 10.1155/2020/1468457

**Published:** 2020-01-30

**Authors:** Peng Wang, Wenbo Liao, Guangru Cao, Yongyan Jiang, Jingcheng Rao, Yi Yang

**Affiliations:** ^1^Department of Orthopaedics, The Affiliated Hospital of Zunyi Medical University, Huichuan District, Dalian Road, Zunyi, Guizhou, China; ^2^Department of Orthopaedics, The Second Affiliated Hospital of Zunyi Medical University, Xinpu New Area, Zunyi, Guizhou, China; ^3^Zunyi Medical University, Huichuan District, Dalian Road, Zunyi, Guizhou, China

## Abstract

**Background:**

Tuberculosis (TB) is an endemic disease in Guizhou. Spinal TB accounts for approximately 50% cases of skeletal TB. The purpose of this study was to investigate the characteristics and management of patients treated for spinal TB in a certain hospital and to provide guidance for the prevention and treatment of spinal TB.

**Methods:**

The clinic records of all patients diagnosed with spinal tuberculosis in a teaching hospital between January 2010 and December 2018 were collected. The epidemiology, clinical characteristics, imaging and laboratory findings, treatment methods, and prognosis were recorded and analyzed.

**Results:**

During this nine-year period, 597 patients with spinal TB were identified. There were 313 males and 284 females with an average age of 43 years. The largest number of patients fell in the age group of 21–30 years; mean time from symptom onset to diagnosis in the hospital was 17 months. Back pain was the main clinical manifestation (89.34%). The most common imaging technique was computed tomography (CT, 96.80%), followed by magnetic resonance imaging (MRI, 84.01%). Majority of the lesions involved the lumbar spine (47.30%), followed by the thoracic spine (40.95%). 178 (29.82%) patients in this study had varying degrees of neurological impairment. 22.78% of the patients selected conservative treatment, and surgical treatment was performed in 483 patients (80.90%).

**Conclusions:**

The incidence of spinal TB was generally on the rise throughout the study period. After diagnosed with spinal TB, all patients got appropriate treatment and achieved good efficacy, but most of the patients did not pay much attention to the disease and receive timely treatment. Thus, it is essential to strengthen the TB preventive strategies, improve the health awareness of residents and universal resident health examination.

## 1. Introduction

As a result of acquired immunodeficiency syndrome (AIDS) and new drug-resistant strains of tuberculosis (TB), the resurgence of spinal TB (STB) has sparked a flurry of activity toward the prevention and treatment of this condition [1]. Despite advances in the methods of diagnosis and treatment, it is still a global public health problem. According to the World Health Organization (WHO), globally, the best estimate is that 10.0 million people (range, 9.0–11.1 million) developed TB in 2017, and the disease burden caused by TB is falling globally, but not fast enough to reach the first (2020) milestones of the End TB Strategy [2]. China's fifth TB epidemiological survey shows that the prevalence of TB in the western region is higher than the central and eastern regions. In Guizhou Province, located in the western region, the economic development is lagging behind, so TB prevention and control started late. Zunyi City has a high incidence and is the key area of prevention and control of TB in Guizhou Province; the total incidence of TB ranked first in the legal infectious diseases because the strategy of the Directly Observed Therapy Shortcourse (DOTS strategy) covering the province was late, leading to poor control and high epidemic of TB. As a common disease of spine, there are a number of publications regarding STB locally and internationally [3,4], but few studies on STB have been conducted in Guizhou. In order to better implement the DOTS strategy, we need to further understand the epidemiological characteristics, diagnosis, and current status of treatment of STB in the region at this moment. This study intends to retrospectively analyze the clinical features, diagnosis, and treatment of 597 cases of patients with STB in a tertiary teaching hospital from January 2010 to December 2018 at Zunyi City, Guizhou Province, and to provide a reference for the prevention and treatment of STB.

## 2. Materials and Methods

We retrospectively reviewed the medical records of patients admitted for STB to the Orthopaedics Department in the Affiliated Hospital of Zunyi Medical University between January 2010 and December 2018 by medical record coding searches. The epidemiology, clinical characteristics, imaging and laboratory findings, treatment methods, and prognosis were recorded. All patients have no autoimmune diseases and non-HIV infected. Diagnosis was established following clinical manifestations and radiological and haematological examination, supplemented by postoperative pathological examination of biopsy specimens. The composite reference standard (CRS) used for categorization of patients into 4 groups: confirmed TB cases (culture positive, smear negative/culture positive, or smear positive/culture positive), probable TB cases (culture negative but showing clinical symptoms, radiological fifindings, and/or histology/cytology suggestive of TB), possible TB cases (negative culture and other tests and only clinical symptoms and/or signs suggestive of TB; in this group, the patient follow-up indicated response to empirical ATT after 3 months), and not TB (culture and all other tests for TB were negative, and patient improved without receiving TB treatment) (Table 1) [5].

## 3. Results

### 3.1. Demographics

The number of patients receiving treatment for STB per year increased after 2010. During this nine-year period, according to the CRS, 597 patients with probable TB were admitted to the Affiliated Hospital of the Zunyi Medical University and categorized into the clinical diagnosis group, which included 313 males (52.43%) and 284 females (47.57%) and their mean age was 43 years (range, 13–89 years) and male to female ratio was 1.10. The largest number of patients fell in the 21–30 years age group (125, 20.94%), followed by 41–50 and 31–40 years age group, accounting for 17.59% and 16.75% of total STB patients, respectively. Mean time from symptom onset to diagnosis in the hospital was 17 months (range, 1 day–240 months) (Table 2).

Risk factors consisted of smoking in 247 patients (41.37%), hypertension in 28 patients (4.69%), hepatitis B in 14 patients (2.35%), and diabetes in 9 patients (1.50%), of which one patient developed diabetic nephropathy. Thirty-one patients had a previous history of TB, including STB (15 cases), pulmonary TB (8 cases), elbow TB (5 cases), and renal TB (3 case). At the time of diagnosis, 183 patients (30.65%) had concomitant pulmonary TB. Thirty-eight cases had other extrapulmonary site involvement, including TB of kidney (15 cases), tuberculous meningitis (8 cases), TB of joint (6 cases), cervical lymph node TB (2 case), epididymis TB (2 cases), tuberculocele (1 case), TB of rib (1 case), fallopian tubes TB (1 case), bladder TB (1 case), and spleen TB (1 case). None of the patients were HIV-positive and immunosuppressing conditions or treatments (e.g., steroid therapy, chemotherapy, etc.).

### 3.2. Clinical Manifestations

Most of the patients were admitted to the hospital because of constitutional symptoms; six patients were diagnosed with STB due to health examination, five patients obtaining medical advice due to pathological fracture caused by spinal TB. Among all the symptoms, back pain was the most common clinical complaint (534, 89.45%), followed by sweating (184, 30.82%), motor weakness (165, 27.64%), numbness (149, 24.96%), weight loss (138, 23.12%), and low-grade fever (133, 22.28%). At physical examination, tenderness was found in 465 patients (77.89%), percussion pain in 420 patients (70.35%) and kyphosis in 183 patients (30.65%). Neurological status was evaluated according to the Frankel classification (Table 3). A total of 178 (29.82%) of the 597 patients had a neurological deficit: Frankel A in 18 patients, Frankel B in 23 patients, Frankel C in 52 patients, and Frankel D in 85 patients (Table 4), of which one patient in Frankel C had a tumor in the infection site, and the pathology after surgery was confirmed as schwannoma.

### 3.3. Imaging Findings

**Figure 1:**
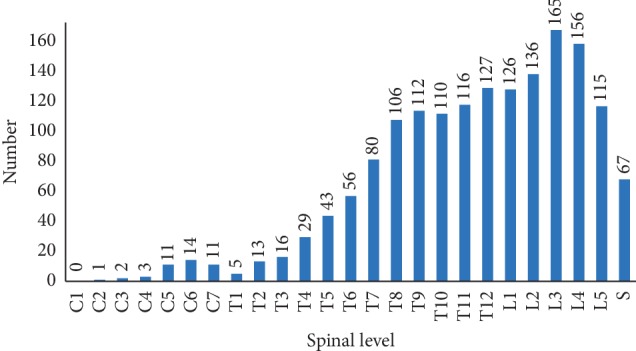
Number of patients with vertebrae involved at each spinal level.

All patients were admitted to the hospital after X-ray examination at the outpatient department. In addition, the most common imaging technique used to evaluate the patients for spinal lesions was CT (96.80%), followed by MRI (84.01%). Radionuclide bone scanning was performed only in 15 patients, all of who had foci of increased uptake. The imaging study consistently suggested that paraspinal abscesses were visible in 439 patients (73.53%). The 597 patients had 1620 lesions, the most commonly involved site was the thoracic vertebral (813, 50.19%), followed by the lumbar vertebral (698, 40.09%), while the sacral vertebral (67, 4.14%) and cervical vertebral (42, 2.59%) were less commonly involved (Figure 1). Approximately, a quarter of patients (145, 24.29%) had three or more vertebral bodies involved, and multiple level skip lesions were seen in 25 (4.19%) cases (Table 5), of which the thoracolumbar spine was the most common site. There were eighteen patients with pathological fracture caused by STB.

### 3.4. Laboratory Test Results

Hemoglobin (normal range is 115–150 g/L) had a routine investigation and in our study ranged from 61 to 164 g/L; the percentage of patients with hemoglobin between 90 and 115 g/L was 44.56%, less than 90 g/L accounting for 5.12%. In addition, the ESR was measured in most patients (578, 96.82%) and ranged from 1 to 140 mm/h.

113 patients had less than 20 mm/h, and 161 patients had greater than 100 mm/h. 563 patients with CRP content ranged from 0.17 to 207.51 mg/l; the CRP was normal in 21.11% of patients, but others increased in varying degrees.

### 3.5. Treatments

**Figure 2:**
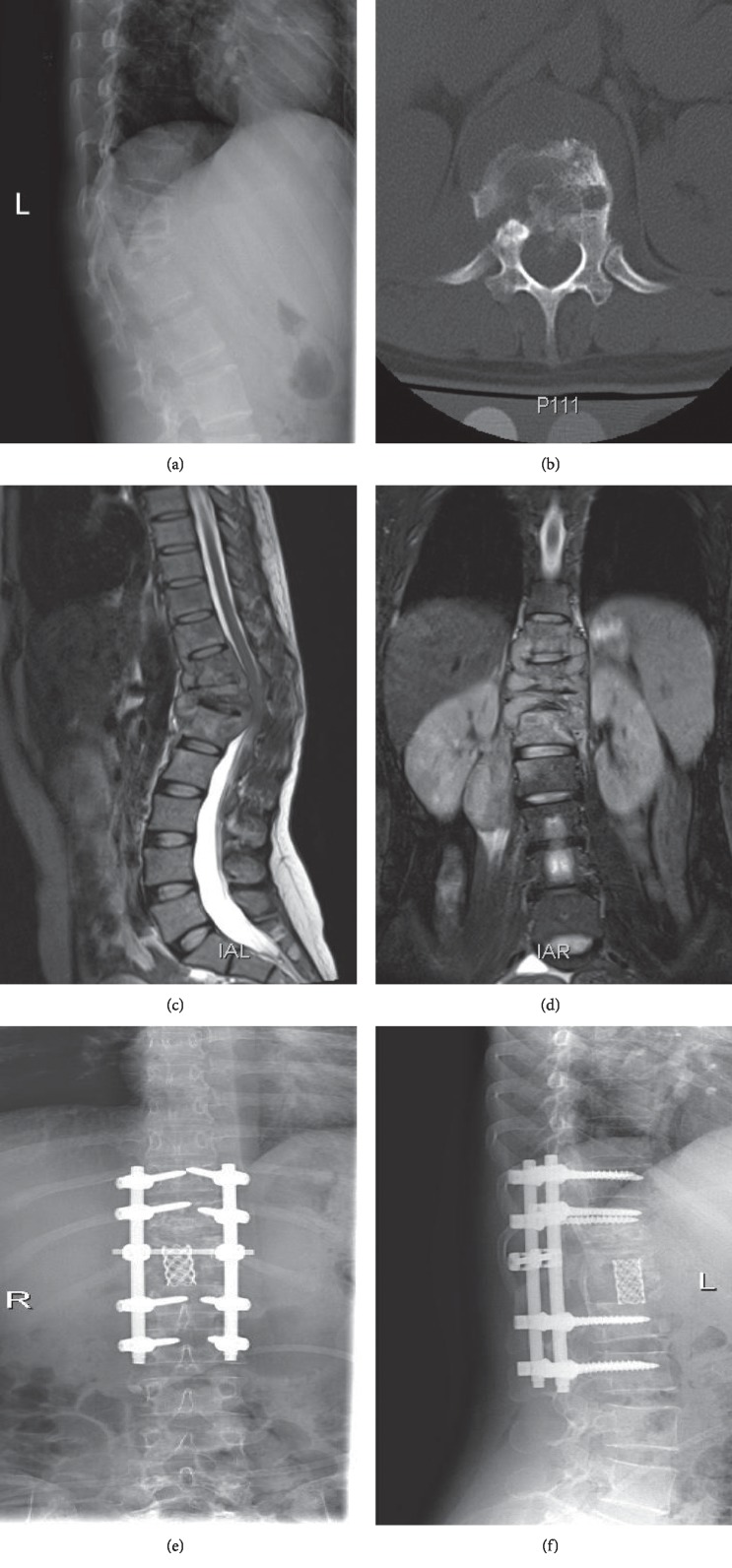
(a) Radiograph of a 32-year-old woman showing T9-L2 vertebral destruction with kyphosis. (b) Axial CT scan showing T12 vertebral destruction. (c) Sagittal MRI showing T9-L2 vertebral destruction with kyphosis, paravertebral, and intraspinal abscesses. (d) Coronal MRI showing T9-L2 vertebral destruction with paravertebral and psoas abscesses. (e, f) A postoperative radiograph shows deformity corrected and satisfactory positioning of the internal fixation device.

Among the 597 patients, 136 patients (22.78%) were chosen for conservative treatment (quadruple antituberculosis agents: isoniazid 0.3 g/d, rifampicin 0.45 g/d, ethambutol 0.75 g/d, and pyrazinamide 0.75 g/d), of which 22 patients were rehospitalized for surgical treatment after antituberculous chemotherapy 2 weeks later. After enough antituberculosis therapy and admission in the hospital, surgical treatment was performed in 483 patients (80.90%) and achieved good efficacy [6] (Figure 2). Anterior debridement was performed in 22 patients (3.69%) with an average length of hospital stay for 25.85 ± 2.3 days. Lateral-anterior debridement, bone grafting, and internal fixation were performed in 261 patients (43.72%) with an average length of hospital stay for 24.11 ± 1.8 days. Posterior debridement, bone grafting, and internal fixation were performed in 90 patients (15.08%) with an average length of hospital stay for 21.37 ± 2.5 days. Anterior debridement, bone grafting, and posterior internal fixation was performed in 86 patients (14.41%) with an average length of hospital stay for 24.24 ± 2.3 days. Among all the patients, there were 24 cases (4.02%) who underwent minimally invasive surgery, anterior or posterior decompression, bone grafting, and posterior internal fixation using the endoscopic system with an average length of hospital stay for 22.00 ± 1.3 days. Of all the surgical patients, two patients were undergoing twice additonal hospitalization and surgery because of the twice internal fixation fracture after the first surgery. Eleven patients were enrolled again after surgery due to sinus formation, of which 3 patients underwent focal debridement and drainage, and 8 patients were healed by strengthening wound dressing replacement.

## 4. Discussion

STB was first described in 1782 by Percival Pott [7]. It accounts for 50% cases of skeletal TB [8]. And 1%–3% of patients suffering from TB have involvement of the skeletal system [9]. Thoracic and lumbar spine is the most common site of involvement, incident in the population of about 3%–5% [10], and is one of the primary causes of spinal deformity and paralysis. As the most common form of skeletal TB, with the increasing incidence of TB, the incidence of STB increased year by year [11]. In our institution, from 2010 to 2018, the number of hospitalized patients with STB showed an overall upward trend. As Shi et al. reported [12], the largest number of patients in our subjects included the 21–30-year-old age group (20.94%). Considering the age of the patients just entered the society, greater mobility, harsh living conditions, and poor health awareness, leading to increased risk of TB infection. At the time of diagnosis, 21.11% of our patients are older than 60 years, which is the reason for the older average age compared to other studies and even have a diagnostic delay for about twenty years [3]. This phenomenon may be closely linked to the characteristics of Guizhou, the socioeconomic status, and poor health consciousness of patients.

STB is characteristically chronic and slowly progressive, more prevalent in immunocompromised persons. Unlike pulmonary TB, STB is seldom accompanied with symptoms such as cough, sputum, fever, body weight loss, or night sweating, which is not easy to attract the attention from patients and doctor and more easily misdiagnosed than pulmonary TB. Similar to other studies [13], back pain was the most common presenting symptom, followed by night sweats, body weight loss, and low-grade fever. Unlike in other studies [3], back pain, weakness, and numbness were the major symptoms. It is noteworthy that 6 patients were diagnosed with STB due to health examination, underwent surgical treatment after enough antituberculous chemotherapy, and recovered well after surgery. Although the number of such patients is less, the importance of health examination in the prevention and treatment of TB, especially in a high incidence area of TB, worthy of further promotion is shown.

Similar to Weng et al.'s study [9], in the current study, concomitant TB of the lung was present in 30.65% of patients. Therefore, people who have pulmonary TB should be strongly alert of the occurrence of spinal TB. In our study, 178 patients (29.82%) had neurological damage such as numbness, weakness, and varying degrees of hypoesthesia. McLain and Isada [14] reported that neurological deficits are common in thoracic and cervical involvement and, if untreated, may progress to complete and incomplete paraplegia, which may be closely related to the structure of the cervical and thoracic spine.

Early diagnosis allows rapid therapeutic intervention and prevention of possible complications. Disk space narrowing and vertebral body destruction are the most common changes seen on plain radiographs, which may however be normal at the earliest stage of the disease. Similar to other studies [4,15], the thoracic and lumbar spines were the spinal segments most commonly affected; in this study, 47.30% involved the lumbar spine and 40.95% involved the thoracic spine. In contrast to the report by Peto et al. [16], 50% of lesions were involved in the thoracic and lumbar spine. MRI and CT are still the most useful modalities for detecting spinal lesions, especially MRI, which has good sensitivity and specificity [17], shows lesions typical of discitis, detects epidural spread, and paraspinal abscesses earlier than other imaging studies [18].

ESR and CRP are the most commonly used parameters to monitor the disease activity and follow up the therapeutic response of STB [19]. ESR is a very sensitive but a highly nonspecific test. In our study, ESR ranged from 1 to 140 mm/h, which was different from other authors' observation [20]. Similarly, the CRP level ranged from 0.17 to 207.51 mg/l; Mulleman et al. [21] pointed out that the CRP level of 23 patients with STBranged from 6 to 197 mg/l. Sudprasert et al. [22] found that the mean value of CRP was 80.4 mg/L in the patients who had neurological deficit due to spinal TB and the earlier declination of CRP in postoperative was closely related to the neurological recovery.

Hemoglobin and albumin are indicators of human nutritional status; malnutrition is one of the important reasons for the development of STB [23]. Of all patients, 297 patients (49.75%) had anemia and 64 patients (10.72%) had hypoproteinemia. Therefore, nutrition support is important in the course of all treatments because STB is a wasting disease [24]. The World Health Organization also recommended the strengthening of nutritional support at the same time of anti-TB treatment [25]. In addition, the occurrence of STB is also closely related to the immune system, and some reports [26] have indicated that immunotherapies that could modulate the immune system and better control M. tuberculosis replication, shorten the course, and significantly improve therapeutic effect in patients with latent TB infection or active disease is a promising approach for host-directed therapy.

The management of STB consists of supportive care, chemotherapy, and surgery. Antituberculous chemotherapy remains the mainstay of therapy throughout the treatment process. The fundamental principle of chemotherapy in TB is that any regime chosen must include multiple drugs and must be given for a prolonged period of time. Therapy should be continued for a duration of 6 to 9 months [27]. Because of its longer cycle and serious adverse reaction, failure of chemotherapy and emergence of drug resistance are frequently due to the failure of compliance [28]. Chemotherapy alone, nevertheless, cannot correct problems arising from bone destruction. Therefore, despite the availability of effective conservative treatments, surgical procedures still assume an important role in the management of STB.

STB patients manifested neurological deficits due to compression of the spinal cord, cauda equina, or nerve root by tuberculous granulation tissue, abscess. Godlwana et al. [29] reported that 56% of their subjects presented with neurological deficits, in which 24% had complete paraplegia and 32% incomplete paraplegia, and the prognosis was closely related to the degree of deficits. 29.82% of patients in this study had varying degrees of neurological impairment. Anti-TB chemotherapy is necessary for surgical intervention for spinal TB. Adequate antitubercular chemotherapy combined with surgical treatment is important to save the spinal cord function and avoid irreversible neurological dysfunction. Surgical treatment can not only decompresses nerves, but also can control the infection, correct deformity, and reshape the stability of the spinal segment [30]. The efficacy of surgical treatment on STB has been confirmed. Anterior, posterior and combined techniques as well as osteotomies, vertebral column resection, have been described to correct spinal alignment and restore sagittal balance [31,32]. The indications for surgery were compression of the spinal cord, cauda equina, or nerve root; severe spinal kyphosis and progressive spinal kyphosis; spine instability; and compression of vital organs by huge abscess. In our population, surgical treatment was performed in 483 patients (80.90%) after anti-TB chemotherapy proved to be effective. All patients' choice of procedure depends on the site and level of vertebral involvement, but generally it is radical lateral-anterior debridement, bone grafting, and internal fixation. At the same time, a part of the patients with STB chose minimally invasive surgical treatment and recovered well; with the development of minimally invasive conceptions, minimally invasive technology has become one of the standard treatment procedures for STB.

## 5. Conclusions

The incidence of STB was generally on the rise throughout the study period. After diagnosed, all patients received appropriate treatment and achieved good efficacy, but most of the patients did not pay much attention to the disease and receive timely treatment. Early diagnosis and prompt treatment of STB are necessary to prevent permanent neurological disability and to minimize spinal deformity. TB remains a major public health problem, and STB should also be widely concerned, especially in Guizhou where the socioeconomic and medical levels are lagging behind; people do not pay much attention to the disease. Thus, it is essential to strengthen the TB preventive strategies and improve the health awareness of residents.

## Figures and Tables

**Table 1:** Algorithm for patient categorization into different categories of the composite reference standard.

CRS category	Result					
	AFB smear	Culture	Symptoms/signs^a^	Radiology^b^	Histology/cytologyun^c^	Follow-up at 3 mo^d^
Confirmed TB	+/−	+	+	+/−	+/−	+

Probable TB	+/−	−	+	+	+	+
	+/−	−	+	+	−	+
	+/−	−	+	−	+	+

Possible TB	+/−	−	+	−	−	+

Not TB	−	−	+	−	−	−

^a^Weight loss, persistent cough, and fever for 2 to 3 weeks. ^b^For radiology, a specimen was positive if the presence of infiltrates or cavities, hilar lymph nodes, pleural effusions, or tuberculomas was noted. ^c^For histology/cytology, a specimen was positive if the presence of caseation necrosis with epitheloid granulomas was reported irrespective of the visual presence or absence of acid-fast bacilli. ^d^For follow-up at 3 months, a specimen was positive if the patient was on antitubercular treatment (ATT) and negative if the patient responded to non-ATT.

**Table 2:** Characteristics of 597 spinal TB patients.

Characteristics	Value
Age (years)	43 (13–89)

Age distribution (years) *N* (%)	
13∼20	55 (9.21)
21∼30	125 (20.94)
31∼40	100 (16.75)
41∼50	105 (17.59)
51∼60	86 (14.07)
61∼70	99 (16.58)
71∼80	23 (3.85)
81∼89	4 (0.67)

Sex, *N* (%)	
Male	313 (52.43)
Female	284 (47.57)
Smoking	247 (41.37)

Comorbidities, *N* (%)	
Pulmonary TB	183 (30.65)
Hypertension	28 (4.69)
Renal TB	15 (2.51)
Hepatitis B	14 (2.35)
Diabetes mellitus	9 (1.50)
Meninges TB	8 (1.34)

Duration of symptoms	17 (1d∼240 m)

**Table 3:** Neurological grades of frankel scale definition.

Frankel A: complete motor and sensory loss below the lesion
Frankel B: incomplete, some sensory loss below the lesion
Frankel C: incomplete, motor and sensory sparing, but the patient is not functional
Frankel D: incomplete, motor and sensory sparing, but patient can stand and walk
Frankel E: normal, complete functional recovery

**Table 4:** Clinical symptoms and signs of 542 spinal TB patients.

Characteristics	Value (%)
Clinical symptoms	
Back pain	534 (89.45)
Night sweats	184 (30.82)
Weakness	165 (27.64)
Numbness	149 (24.96)
Weight loss	138 (23.12)
Fever	133 (22.28)

Clinical signs	
Tenderness	465 (77.89)
Percussion pain	420 (70.35)
Kyphosis	183 (30.65)

Frankel classification, *N* (%)	
A	18 (3.01)
B	23 (3.85)
C	52 (8.71)
D	85 (14.24)

**Table 5:** Imaging characteristics and laboratory findings of 597 spinal TB patients.

Characteristics	Value, *n* (%)
Location	
Cervical	20 (2.70)
Thoracic	303 (40.95)
Lumbar	350 (47.30)
Sacral	67 (9.05)

Vertebra involved	
1	92 (15.41)
2	360 (60.30)
≥3	145 (24.29)

Skipped lesion	
C, T	2 (0.34)
T, L	13 (2.18)
T, T	4 (0.67)
L, S	3 (0.50)
T, L, S	3 (0.50)

(C-cervical vertebral; T-thoracic vertebral; L-lumbar vertebral; S-sacral vertebral).

## Data Availability

The data used to support the findings of this study are available from the corresponding author upon request.
